# Spontaneous splenic rupture as a rare complication of *Plasmodium vivax*: a case report

**DOI:** 10.11604/pamj.2026.53.3.50377

**Published:** 2026-01-05

**Authors:** Meriem Mouharir, Badr Jouabri, Zakaria Chahbi, Mohamed Fassi Fihri, Hicham Baba, Said Kaddouri, Rachid Elbarni, Hassan Qacif, Mohammed Zyani

**Affiliations:** 1 Department of Internal Medicine, Avicenne Military Hospital, Marrakech, Morocco,; 2 Department of General Surgery, Avicenne Military Hospital, Marrakech, Morocco

**Keywords:** Malaria, spontaneous splenic rupture, conservative treatment, case report

## Abstract

Spontaneous splenic rupture is a rare but potentially fatal condition. We report the case of a thirty-year-old Moroccan soldier who presented with fever six months after returning from a deployment in a malaria-endemic country, despite appropriate mefloquine chemoprophylaxis. Malaria due to Plasmodium vivax was confirmed by blood film, and oral treatment (Artemether-Lumefantrine) was initiated. Within twenty-four hours of admission, the patient developed acute abdominal pain with hemodynamic instability and a decrease in haemoglobin level. Abdominal computed tomography revealed a subcapsular splenic haematoma with moderate haemoperitoneum, consistent with spontaneous splenic rupture. A decision of conservative management was established with bed rest, close haemodynamic monitoring, intravenous rehydration solution and blood transfusion as necessary, without splenectomy. The outcome was favourable, characterised by defervescence, complete resolution of abdominal pain and no further decrease of haemoglobin. This case illustrates that splenic rupture should be considered in malaria patients with abdominal pain associated with clinical features of hypovolaemia and no history of trauma. It also shows that conservative management can preserve splenic function and lead to a good clinical outcome.

## Introduction

Spontaneous splenic rupture is an uncommon but potentially life-threatening complication, mainly owing to late recognition and delayed treatment. Among tropical infections, malaria is the principal cause of non-traumatic splenic rupture. Several published reports have particularly implicated *Plasmodium vivax*, in which the acute phase is characterized by rapid splenic enlargement and increased capsular fragility, thereby predisposing the spleen to rupture. This case report describes a rare presentation of non-traumatic splenic rupture during *Plasmodium vivax* malaria in a thirty-year-old soldier six months after returning from a malaria-endemic area. Highlighting the importance of early diagnosis and adequate management of this unusual complication.

## Patient and observation

**Patient information:** we report the case of a 30-year-old Moroccan soldier with no notable past medical history. Presented to our department with fever and chills, but no bowel habit change. He had previously resided in Congo, where he received during his stay a malaria chemoprophylaxis based on mefloquine and reported good adherence. Symptoms began six months after his return.

**Clinical findings:** initial physical examination revealed pale conjunctivae with normal vital signs: blood pressure: 135/91 mmHg, pulse rate: 80 beats per minute, respiratory rate: 21 breaths per min and Temperature: 37.3°C. Abdominal examination revealed diffuse direct tenderness without guarding. The patient reported no history of abdominal or chest trauma.

**Figure 1:**
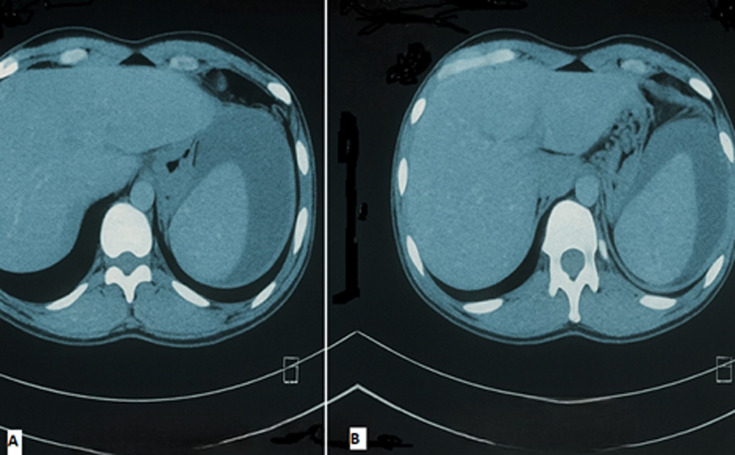
A, B) computed tomography scan of the abdomen with IV contrast showing an enlarged spleen with subcapsular splenic haematoma compressing the splenic parenchyma, consistent with spontaneous splenic rupture

**Diagnostic assessment:** initial blood investigations showed anaemia with hemoglobin level of 10.5 g/dl with thick and thin blood smear positive for *Plasmodium vivax* with +2 parasitemia. The diagnosis of malaria was established and oral anti-malarial treatment (Artemether-Lumefantrine) was initiated. Within 48 hours of admission, the patient developed acute abdominal pain with abdominal guarding and hemodynamic instability. Laboratory findings showed a decrease in hemoglobin to 8.S5 g/dl. An urgent abdominopelvic computerized tomography (CT) scan showed a subcapsular splenic hematoma (Figure 1) with a moderate abdominal effusion.

**Diagnosis:** non-traumatic rupture of the spleen and hemoperitoneum due to *Plasmodium vivax* malaria.

**Therapeutic interventions:** the patient was treated successfully with a non-operative approach. This conservative treatment consists of bed rest in the hospital, rigorous haemodynamic and clinical surveillance, with intravenous fluid and blood transfusion. After completing a full course of antimalarial therapy, the patient was discharged on the 15^th^ day of his hospital stay with scheduled follow-up appointments.

**Follow-up and outcome of interventions:** he showed progressive improvement. He was asymptomatic, physical examination was normal. A follow-up CT scan demonstrated a regressing subcapsular splenic haematoma without an increase in the amount of intraperitoneal fluid.

**Patient perspective:** the patient expressed satisfaction with the medical care and follow-up he received. He specifically reported being pleased that the medical team opted for conservative management instead of surgery.

**Informed consent:** written informed consent was obtained from the patient for publication of this case report and accompanying clinical information.

## Discussion

Spontaneous splenic rupture is a rare clinical entity. In the context of malaria, most reported cases occur during the acute phase of infection and are associated with *Plasmodium vivax*, although rare cases related to other *Plasmodium* species have been reported [1,2]. Several pathophysiological mechanisms have been proposed to explain spontaneous splenic rupture in malaria. First, diffuse cellular hyperplasia and vascular congestion within the spleen increase intrasplenic tension. Second, external splenic compression due to raised intra-abdominal pressure during activities such as sneezing, coughing or defecation. Third, reticuloendothelial hyperplasia can promote venous congestion, thrombosis and infarction, leading to subcapsular bleeding and, ultimately, disruption of the splenic capsule [1-3]. This life-threatening complication is often underdiagnosed and underreported [4], even occurring after treatment initiation. Clinicians should consider this diagnosis in malaria patients presenting with abdominal pain, fever, and progressive anaemia with or without haemodynamic instability and no history of trauma [5,6].

The diagnostic work-up should begin with a complete blood count, since thrombocytopenia and anaemia are commonly observed [3]. Radiological assessment is then required, with contrast-enhanced abdominal CT regarded as the gold-standard modality [3,5]. This examination, however, is usually limited to haemodynamically stable patients. Classically, splenectomy was considered the standard treatment for malarial splenic rupture [2,5]. More recent reports and small series, however, indicate that conservative management can be safe and effective in carefully selected haemodynamically stable patients [6,7]. Non-operative management typically consists of maintaining the patient on strict bed rest, providing intravenous fluids, and ensuring close monitoring of vital signs and abdominal findings, with serial measurements of haemoglobin and repeat imaging, in addition to appropriate antimalarial therapy [7]. In our patient, this conservative strategy resulted in a favourable outcome. This case illustrates that a spleen-preserving approach may be considered, even in patients with haemodynamic instability, provided that stabilisation is achieved and careful surveillance is possible. Importantly, this strategy can only be safely implemented in settings where continuous monitoring is available, and a surgical team is immediately on hand to perform splenectomy if the patient´s condition deteriorates.

## Conclusion

Spontaneous splenic rupture in malaria is a serious complication. Management strategies are guided by the patient’s haemodynamic status and the extent of splenic injury. Conservative treatment is a reasonable option in haemodynamically stable patients, whereas splenectomy remains the treatment of choice in those with significant haemoperitoneum and persistent instability. Spontaneous splenic rupture should be suspected in any patient with malaria who presents with acute abdominal symptoms and fever, particularly in an endemic context. Any delay in diagnosis and intervention is likely to worsen the prognosis.
